# Editorial: Stimuli-Responsive Nanoparticles for Anti-cancer Therapy

**DOI:** 10.3389/fbioe.2021.797619

**Published:** 2021-11-15

**Authors:** Saji Uthaman, In-Kyu Park, Kang Moo Huh, James Lai, Mitsuhiro Ebara

**Affiliations:** ^1^ Chemical and Biological Engineering, IOWA State University, Ames, IA, United States; ^2^ Department of Biomedical Sciences, Chonnam National University, Gwangju, South Korea; ^3^ Department of Polymer Science and Engineering, Chungnam National University, Daejeon, South Korea; ^4^ Department of Bioengineering, University of Washington, Washington, ME, United States; ^5^ International Center for Materials Nanoarchitectonic, National Institute for Materials Science, Tsukuba, Japan

**Keywords:** nanoparticle, drug delivery, photothermal therapy, molecular Imaging, ultra sound

The Research topic entitled “Stimuli-Responsive Nanoparticles for Anti-Cancer Therapy” addresses the current advances in the stimuli-responsive nanoparticles for anti-cancer. This issue comprises nine selected peer-reviewed manuscripts (original research and review articles) discussing the latest updates on various stimuli-responsive nanoparticles used in oncology. Different types of nanoparticles, including activated polymeric delivery systems (Peng et al.), multifunctional magnetic nanobubbles (Jin et al.), folic acid functionalized gelatin–AuNPs composite scaffolds (Chen et al.), Zinc oxide nanocrystals (Racca et al.), and Near-Infrared responsive Phase-shifting nanoparticles (Xu et al.) are presented through original research works. These novel nanoparticles with tailor-made properties offer a universal approach for anti-cancer therapy as their responsiveness depends on the general physiological properties commonly found in all tumors.

The first example of external stimuli-responsive nanoparticles is presented by (Racca et al.). In this study, amino-propyl functionalized ZnO nanocrystals (ZnO NCs) combined with ultrasound shock waves (SW) were used to treat cancer cells. The ZnO NCs demonstrated synergism in combination with SW stimulus. In another study by Jin et al., multifunctional magnetic nanobubbles (MF-MNBs) comprising of poly (D, L-lactide-co-glycolide (PLGA) - polyethylene glycol–folate (PLGA-PEG-FA) polymer-based nanobubbles were evaluated as tumor-targeted ultrasound (US)/magnetic resonance (MR) imaging and focused ultrasound (FUS)-triggered drug delivery system. The MF-MNB exhibited ligand-receptor mediated tumor accumulation and focused ultrasound FUS-triggered drug delivery for efficient cancer treatment. Chen et al. have demonstrated photothermal ablation using near-infra-red irradiation as an external stimulus for killing cancer cells. This study synthesized folic acid (FA)-functionalized composite scaffold by hybridizing FA-conjugated gelatin and FA-modified AuNPs and using ice particulates as porogen material. *In vitro* and *In vivo* studies demonstrated that FA-functionalized gelatin–AuNPs composite scaffolds could elicit local photothermal ablation of breast cancer cells. Next, near-infrared responsive phase-shifted nanoparticles (NRPNs) have been designed by Xu et al. for magnetically targeted MR/US imaging and photothermal therapy of tumors. The near-infrared responsive phase-shifted nanoparticles (NRPNs) comprise PLGA nanoparticles encapsulated with indocyanine green (ICG), magnetic Fe_3_O_4_ nanoparticles, and perfluoro pentane (PFP). Upon irradiating with a NIR laser, the NRPNs, a phase-shifted expansion effect due to the quick conversion from light to heat by ICG and Fe_3_O_4_, can be used for ultrasound (US) imaging. In another study, Peng et al. have reported on an activated nanoparticle system comprising of poly (D, L-lactide-co-glycolide; PLGA)-containing iron oxide nanoparticles (IOs) for biological imaging, using fucoidan/hyaluronic acid (FU/HA) to achieve targeting activity and applying polyethylene glycol-modified gelatin (PG)-carrying a phytochemical, epigallocatechin gallate (EGCG) to eradicate prostate tumors. This study demonstrated that the combination of therapeutic and molecular imaging could effectively target prostate cancer cells.

Besides the original research articles, this research topic also has a series of review articles that summarized the recent advances in anti-cancer therapy using external and internal stimuli-responsive metallic nanoparticles (Mohapatra et al.), pathological pH-responsive polymeric nano biosensors (Kumar et al.), tumor microenvironment responsive nanoparticles (Thomas et al.), and plant virus nanoparticles (Hefferon et al.). As a starting example, Kumar et al. have summarized the recent developments in the design, preparation, and characterization of pH-responsive nanobiosensors and their ability to behave as efficient *in vivo* nano theranostics agents in acidic cancer environments. Thomas et al. have summarized the different types of internal (pH, redox, enzyme, ROS, hypoxia) stimuli-responsive nanoparticle drug delivery systems, Mohapatra et al., have outlined the role of different metallic nanotherapeutics in anti-cancer therapy, as well as their combinational effects with multiple stimuli for enhanced anti-cancer treatment. Finally, Hefferon et al. explore plant viruses as epitope-carrying nanoparticles and novel tools in cancer immunotherapy.

To summarize, we hope that this research topic will provide insights into the recent trends in nanomedicine, especially in oncology, using stimuli-responsive nanoparticles, providing insights into the development of targeted nanomedicine. The editors hope that the Research Topic “Stimuli-Responsive Nanoparticles for Anti-Cancer Therapy” will contribute to the progress of research and development activities in the field of nanomedicine, inspiring and offering a universal approach for anti-cancer therapy by taking advantage of the physiological properties commonly found in all tumors.

